# Effect of Leptin Deficiency on the Skeletal Response to Hindlimb Unloading in Adult Male Mice

**DOI:** 10.1038/s41598-019-45587-0

**Published:** 2019-06-27

**Authors:** Jessica A. Keune, Adam J. Branscum, Carmen P. Wong, Urszula T. Iwaniec, Russell T. Turner

**Affiliations:** 1 Skeletal Biology Laboratory, School of Biological and Population Health Sciences, Oregon State University, Corvallis, OR 97331 USA; 2 Biostatistics Program, School of Biological and Population Health Sciences, Oregon State University, Corvallis, OR 97331 USA; 3 Center for Healthy Aging Research, Oregon State University, Corvallis, OR 97331 USA

**Keywords:** Physiology, Endocrinology

## Abstract

Based on body weight, morbidly obese leptin-deficient *ob/ob* mice have less bone than expected, suggesting that leptin plays a role in the skeletal response to weight bearing. To evaluate this possibility, we compared the skeletal response of wild type (WT) and *ob/ob* mice to hindlimb unloading (HU). Mice were individually housed at 32 °C (thermoneutral) from 4 weeks of age (rapidly growing) to 16 weeks of age (approaching skeletal maturity). Mice were then randomized into one of 4 groups (n = 10/group): (1) WT control, (2) WT HU, (3) *ob/ob* control, and (4) *ob/ob* HU and the results analyzed by 2-way ANOVA. *ob/ob* mice pair-fed to WT mice had normal cancellous bone volume fraction (BV/TV) in distal femur, lower femur length and total bone area, mineral content (BMC) and density (BMD), and higher cancellous bone volume fraction in lumbar vertebra (LV). HU resulted in lower BMC and BMD in total femur, and lower BV/TV in distal femur and LV in both genotypes. Cancellous bone loss in femur in both genotypes was associated with increases in osteoclast-lined bone perimeter. In summary, leptin deficiency did not attenuate HU-induced osteopenia in male mice, suggesting that leptin is not required for bone loss induced by unweighting.

## Introduction

Leptin, a hormone produced primarily by adipocytes, serves as a messenger in a feedback loop between adipose tissue and the hypothalamus and contributes to regulation of energy intake and energy expenditure^1,2^. *ob/ob* mice, homozygous for loss of function mutation in the obesity gene (*ob*), are unable to generate leptin^1,2^. *ob/ob* mice become morbidly obese due to the combined influence of increased food intake and decreased energy expenditure^1^.

The physiological actions of leptin extend beyond energy metabolism and include regulation of skeletal growth and maturation^3–6^; a detailed review of the effects of leptin on the skeleton can be found in Reid *et al*.^7^ Leptin stimulates endochondral ossification and osteoblast differentiation and activity in long bones of the lower limb, where the hormone also inhibits differentiation of bone marrow mesenchymal stem cells to adipocytes^8–10^. Compared to wild type (WT) mice, *ob/ob* mice have smaller craniofacial dimensions^11^, shorter long bones^12,13^ and, when evaluated by dual energy x-ray absorptiometry (DXA), have lower total body and femur bone area, bone mineral content (BMC) and bone mineral density (BMD)^8^. Based on microcomputed tomographic (µCT) and histomorphometric analyses, *ob/ob* mice have bone- and bone compartment-specific alterations in cortical and cancellous bone mass and architecture^8,14^, and greatly increased levels of marrow adipose tissue (MAT)^15,16^. Whereas, total and cortical bone mass is normal or lower in *ob/ob* mice, cancellous bone mass, depending upon skeletal site and age, can be lower, normal, or higher^7^. Hypothalamic leptin gene therapy reverses skeletal abnormalities in *ob/ob* mice^17^. While the literature strongly supports an important role of leptin in increasing bone accrual prior to peak bone mass and in maintaining normal bone turnover, the hormone likely has additional, less well characterized, actions on bone metabolism. In this regard, there is conflicting evidence that leptin plays a role in mediating skeletal response to changes in body weight.

Kapur *et al*. reported leptin signaling to be a negative modulator of bone mechanosensitivity^18^. Specifically, a loading strain of ∼2,500 μϵ, which was insufficient to produce a bone formation response in B6 mice, significantly increased bone formation parameters in leptin-deficient *ob*/*ob* mice. However, other studies suggest that leptin is required for full expression of positive effects of weight on bone mass. For example, total femur mass is positively associated with body weight in WT mice^19^. Also, mice heterozygous for the *ob* gene (*ob*/+ mice) have adipocytes with a reduced capacity to generate leptin but have near normal leptin levels due to compensatory increases in white adipose tissue (WAT) mass^19^. *ob*/+ mice exhibit an association between body weight and bone mass nearly identical to WT mice. In contrast, weight differences in *ob/ob* mice have a positive but quantitatively smaller effect on bone mass^20^. Thus, it is possible that leptin is not required for skeletal adaptation to changes in weight but the hormone may enhance the magnitude of response by increasing sensitivity of bone to external loads.

If leptin enhances the skeletal response to changes in magnitude of ground reaction forces during weight bearing, leptin-deficient *ob/ob* mice would be predicted to exhibit an attenuated bone response to reduced skeletal loading. To test this possibility, we compared the skeletal response of male WT and *ob/ob* mice housed at thermoneutral to hindlimb unloading (HU), a ground-based model for microgravity^21^. HU unweights the hindlimbs, facilitating investigation of the role of leptin in the skeletal response to changes in weight^21^. The study was performed at thermoneutral because leptin plays an important role in thermoregulation and mild cold stress induced by conventional room temperature housing results in rapid premature cancellous bone loss in mice^22,23^. Data were analyzed by 2-way analysis of variance to establish the main effects (genotype and skeletal loading status) and their interaction.

## Results

*ob/ob* mice were pair fed to WT mice from 4 to 16 weeks of age and all mice were pair fed to WT HU mice during HU. Food intake averaged 2.3 ± 0.0 g/d for two weeks prior to HU and 1.8 ± 0.1 g/d during HU (Supplemental Fig. S1). As expected (because of pair feeding), significant genotype, HU, or interaction effects were not detected for food intake.

**Figure 1:**
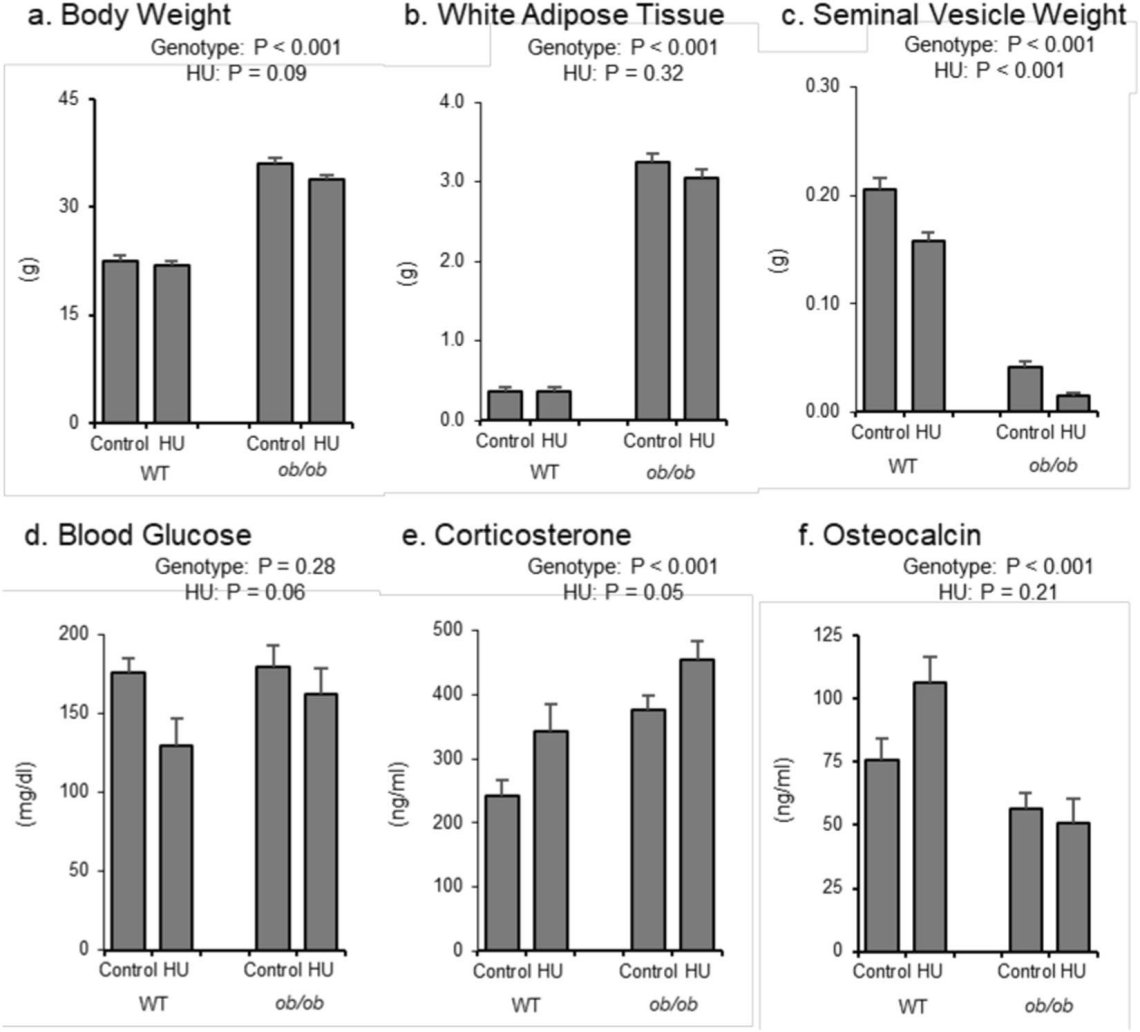
Effects of genotype and hindlimb unloading (HU) on (**a**) body weight, (**b**) abdominal white adipose tissue (WAT) weight, (**c**) seminal vesicle weight, (**d**) blood glucose, (**e**) serum corticosterone, and (**f**) serum osteocalcin in WT and *ob/ob* mice. Statistical analysis: Two-way ANOVA. P-values for main effects of leptin status (genotype) and skeletal loading status (HU) significant at P ≤ 0.05. Significant (P ≤ 0.05) genotype x HU interactions were not detected with treatment for any of the endpoints evaluated. Mean ± SEM; N = 10/group.

The respective and combined effects of leptin status and HU on body weight, abdominal WAT weight, seminal vesicle weight (an index of gonadal hormone status), blood glucose levels, and serum corticosterone and osteocalcin levels are shown in Fig. 1. Compared to WT mice, *ob/ob* mice had greater body weight (Fig. 1a), abdominal WAT weight (Fig. 1b), and corticosterone levels (Fig. 1e), lower seminal vesicle weight (Fig. 1c) and osteocalcin levels (Fig. 1f), and no difference in glucose levels (Fig. 1d). No significant difference in WAT weight was observed in response to HU but HU resulted in trends for lower body weight (p = 0.09) and blood glucose levels (p = 0.06). HU resulted in lower seminal vesicle weight and higher corticosterone levels. No significant genotype by treatment interaction was detected for any of the measured endpoints.

**Figure 2:**
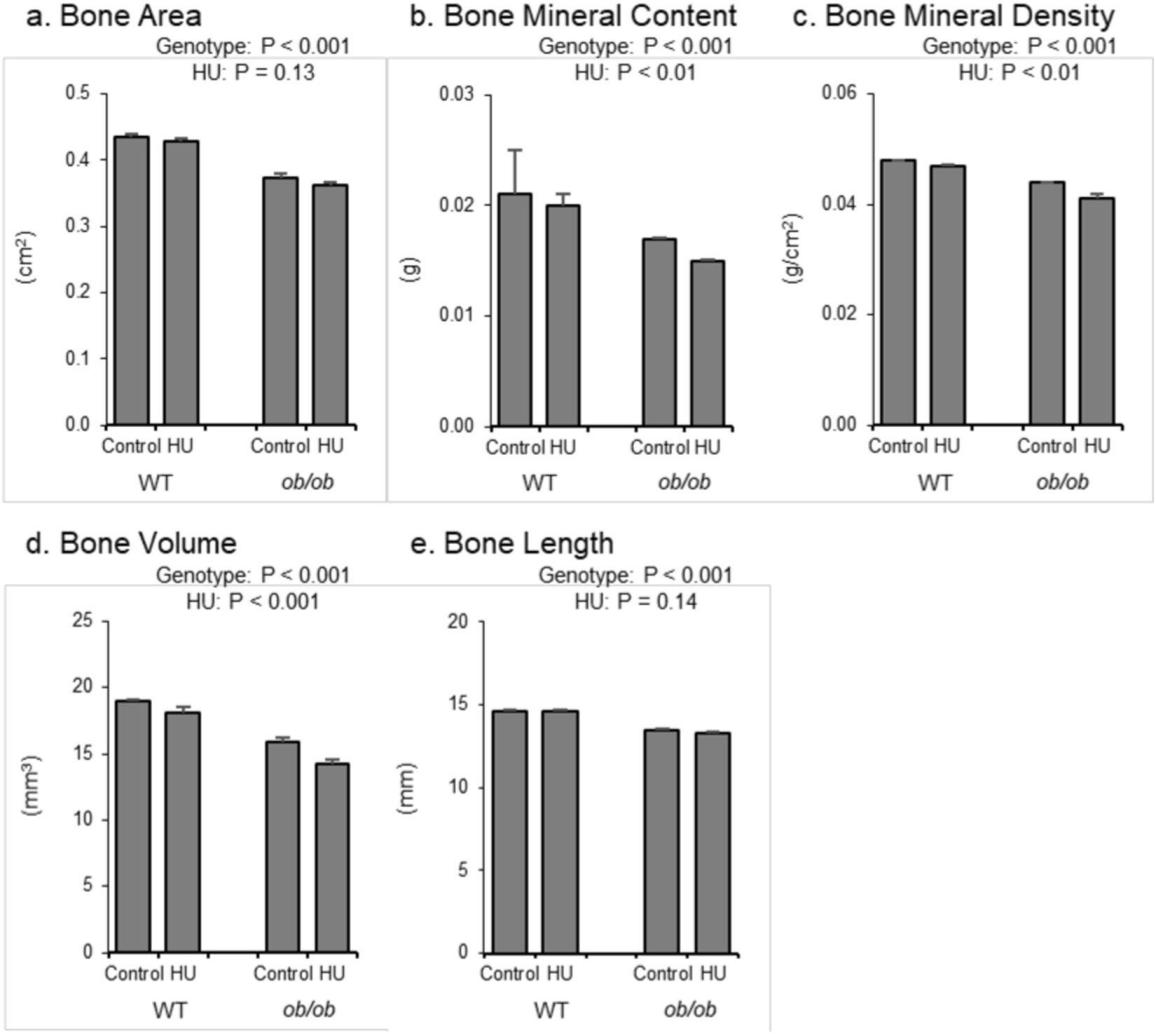
Effects of genotype and hindlimb unloading (HU) on total femur in WT and *ob/ob* mice. Shown are (**a**) bone area, (**b**) bone mineral content (BMC), (**c**) bone mineral density (BMD), (**d**) bone volume, and (**e**) bone length. Statistical analysis: Two-way ANOVA. P-values for main effects of leptin status (genotype) and skeletal loading status (HU) significant at P ≤ 0.05. Significant (P ≤ 0.05) genotype x HU interactions were not detected with treatment for any of the endpoints evaluated. Mean ± SEM; N = 10/group.

The respective and combined effects of leptin status and HU on total femur are shown in Fig. 2. *ob/ob* mice had lower total femur bone area (Fig. 2a), bone mineral content (Fig. 2b), bone mineral density (Fig. 2c), bone volume (Fig. 2d), and bone length (Fig. 2e) than WT mice. No significant difference in total femur bone area or length was observed in response to HU. However, HU mice had lower total femur bone mineral content, bone mineral density, and bone volume than control mice. No significant genotype by treatment interaction was detected for any of the measured endpoints.

**Figure 3:**
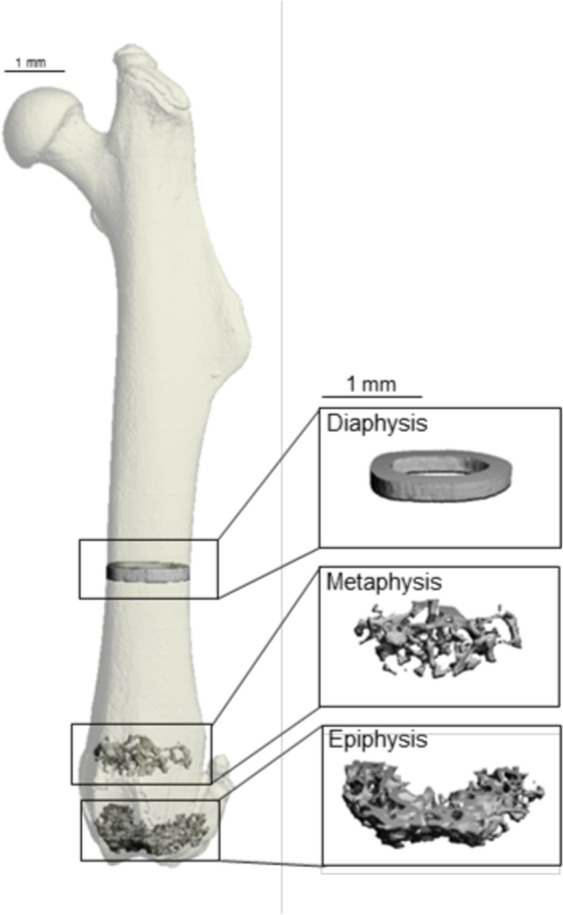
Volume of interest for the femur diaphysis, metaphysis and epiphysis. Image compiled by J.A.K.

Following evaluation of total femur, bone architecture was assessed in the femur diaphysis, distal femur metaphysis, and distal femur epiphysis. The regions of interest are illustrated in Fig. 3.

**Figure 4:**
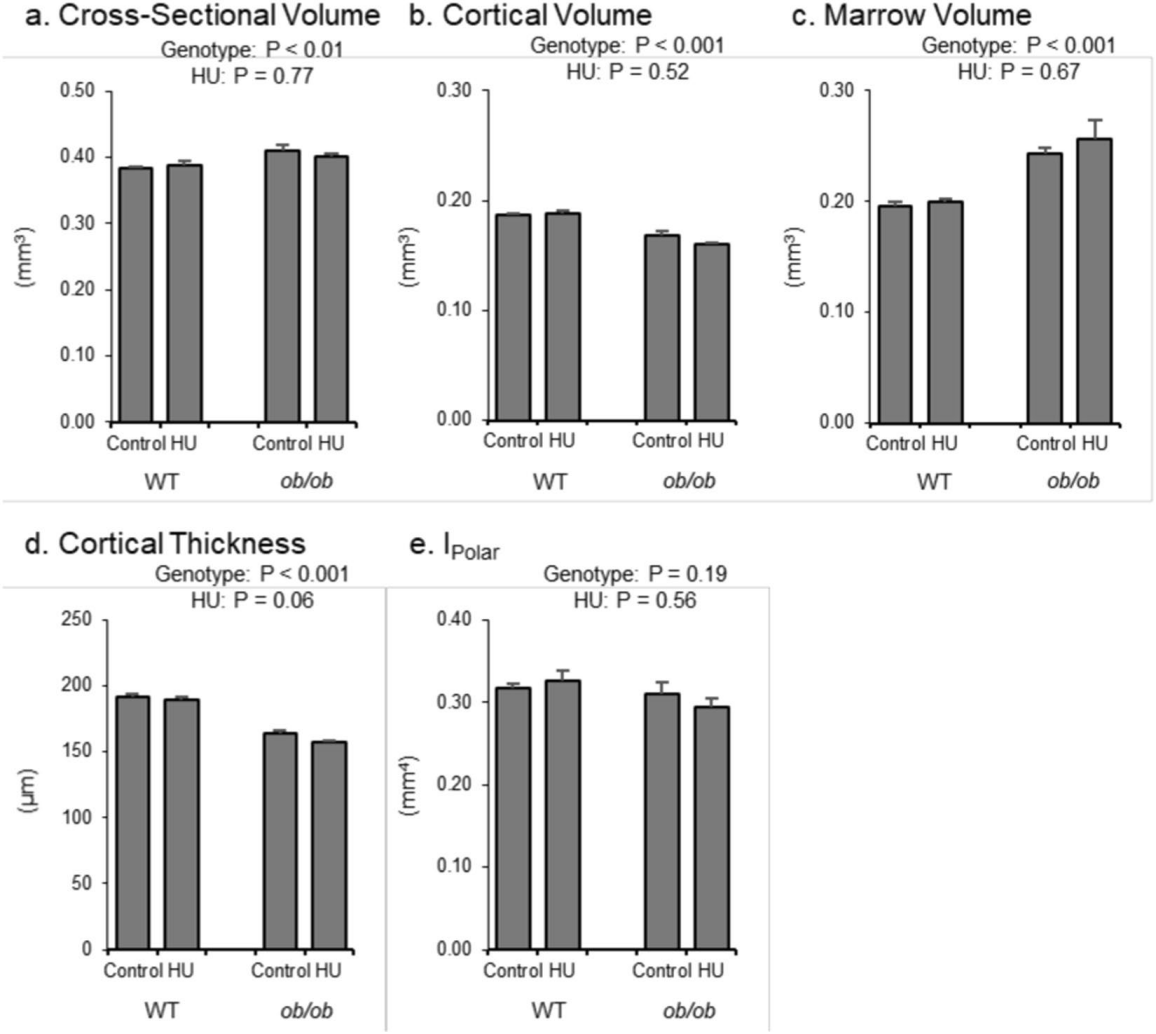
Effects of genotype and hindlimb unloading (HU) on cortical bone microarchitecture in the femur diaphysis in WT and *ob/ob* mice. Shown are (**a**) cross-sectional volume, (**b**) cortical volume, (**c**) marrow volume, (**d**) cortical thickness, and (**e**) polar moment of inertia (I_Polar_). Statistical analysis: Two-way ANOVA. P-values for main effects of leptin status (genotype) and skeletal loading status (HU) significant at P ≤ 0.05. Significant (P ≤ 0.05) genotype x HU interactions were not detected with treatment for any of the endpoints evaluated. Mean ± SEM; N = 10/group.

The respective and combined effects of leptin status and HU on cortical bone microarchitecture in the femur diaphysis are shown in Fig. 4. *ob/ob* mice had greater cross-sectional volume (Fig. 4a) and marrow volume (Fig. 4c) but lower cortical volume (Fig. 4b) and cortical thickness (Fig. 4d) than WT mice. No significant difference in cross-sectional volume or cortical volume was observed in response to HU but cortical thickness tended (p = 0.06) to be lower with HU. I_Polar_ (Fig. 4e) was not different between genotypes and not altered by HU. No significant genotype by treatment interaction was detected for any of the measured endpoints.

**Figure 5:**
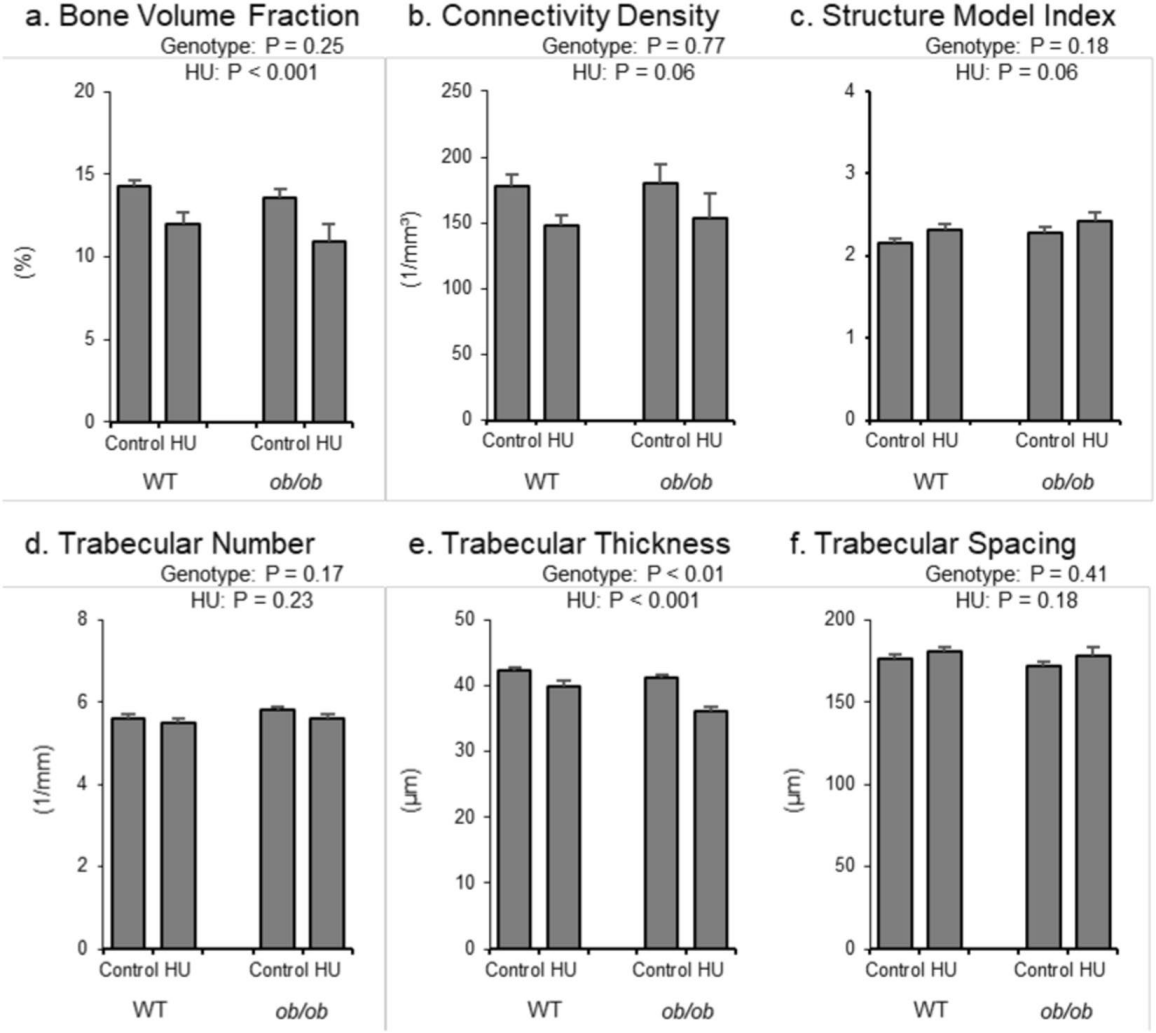
Effects of genotype and hindlimb unloading (HU) on cancellous bone microarchitecture in the distal femur metaphysis in WT and *ob/ob* mice. Shown are (**a**) cancellous bone volume fraction, (**b**) connectivity density, (**c**) structure model index, (**d**) trabecular number, (**e**) trabecular thickness, and (**f**) trabecular spacing. Statistical analysis: Two-way ANOVA. P-values for main effects of leptin status (genotype) and skeletal loading status (HU) significant at P ≤ 0.05. Significant (P ≤ 0.05) genotype x HU interactions were not detected with treatment for any of the endpoints evaluated. Mean ± SEM; N = 10/group.

The respective and combined effects of genotype and HU on cancellous bone microarchitecture in the distal femur metaphysis are shown in Fig. 5. Bone volume fraction (Fig. 5a), connectivity density (Fig. 5b), structure model index (Fig. 5c) trabecular number (Fig. 5d), and trabecular spacing (Fig. 5f) were not different between genotypes. However, *ob/ob* mice had lower trabecular thickness than WT mice (Fig. 5e). HU resulted in lower bone volume fraction, lower trabecular thickness, and a tendency (p = 0.06) for lower connectivity density and structure model index. No significant difference in trabecular number or spacing was observed in response to HU. No significant genotype by treatment interaction was detected for any of the measured endpoints.

**Figure 6:**
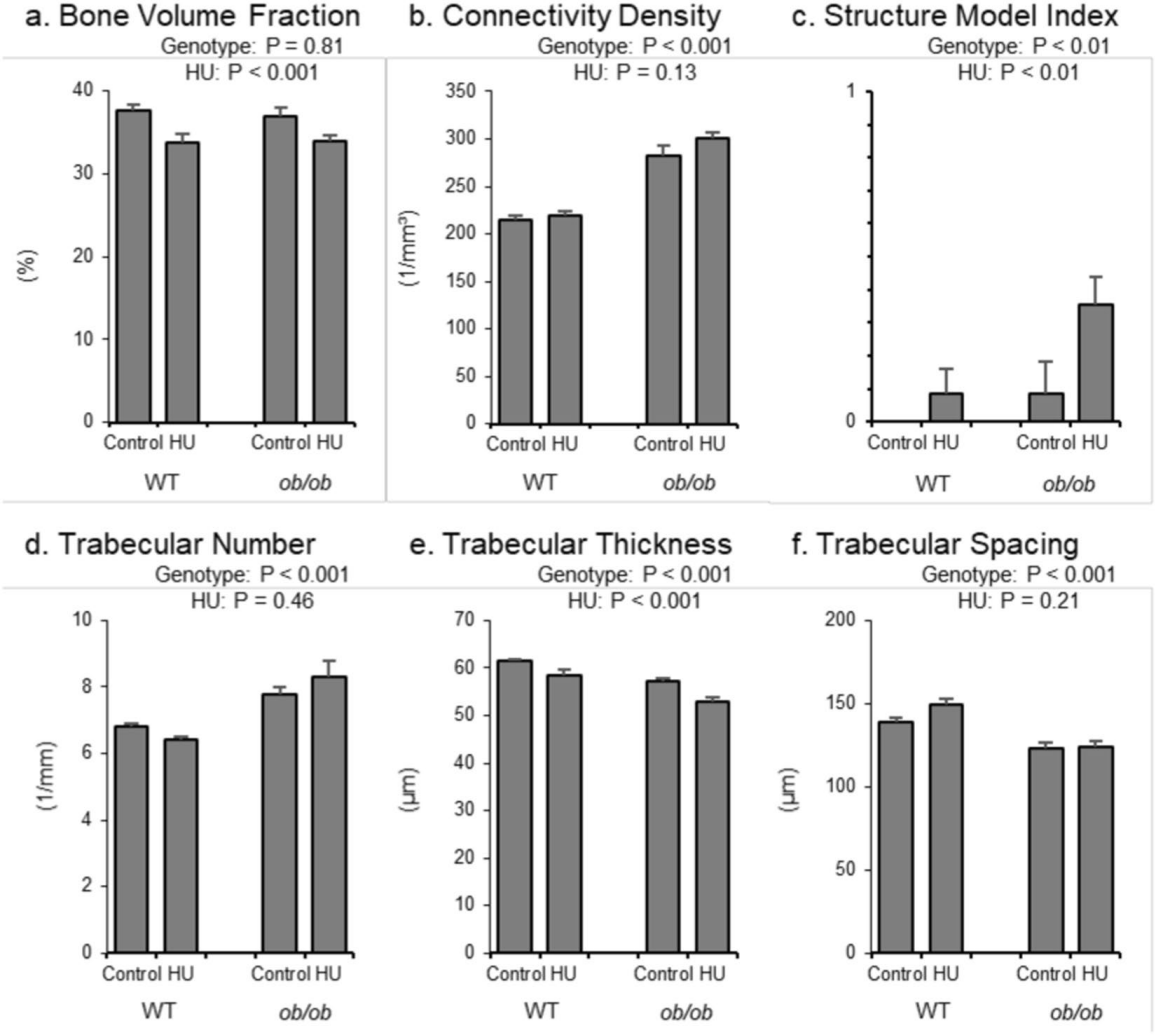
Effects of genotype and hindlimb unloading (HU) on cancellous bone microarchitecture in the distal femur epiphysis in WT and *ob/ob* mice. Shown are (**a**) cancellous bone volume fraction, (**b**) connectivity density, (**c**) structure model index, (**d**) trabecular number, (**e**) trabecular thickness, and (**f**) trabecular spacing. Statistical analysis: Two-way ANOVA. P-values for main effects of leptin status (genotype) and skeletal loading status (HU) significant at P ≤ 0.05. Significant (P ≤ 0.05) genotype x HU interactions were not detected with treatment for any of the endpoints evaluated. Mean ± SEM; N = 10/group.

The respective and combined effects of genotype and HU on cancellous bone microarchitecture in the distal femur epiphysis are shown in Fig. 6. Bone volume fraction was not different between genotypes (Fig. 6a). However, *ob/ob* mice had greater connectivity density (Fig. 6b), structure model index (Fig. 6c), and trabecular number (Fig. 6d), and lower trabecular thickness (Fig. 6e) and trabecular spacing (Fig. 6f) than WT mice. HU resulted in lower bone volume fraction and trabecular thickness and higher structure model index, but no significant difference in connectivity density, trabecular number or trabecular spacing. No significant genotype by treatment interaction was detected for any of the measured endpoints.

**Figure 7:**
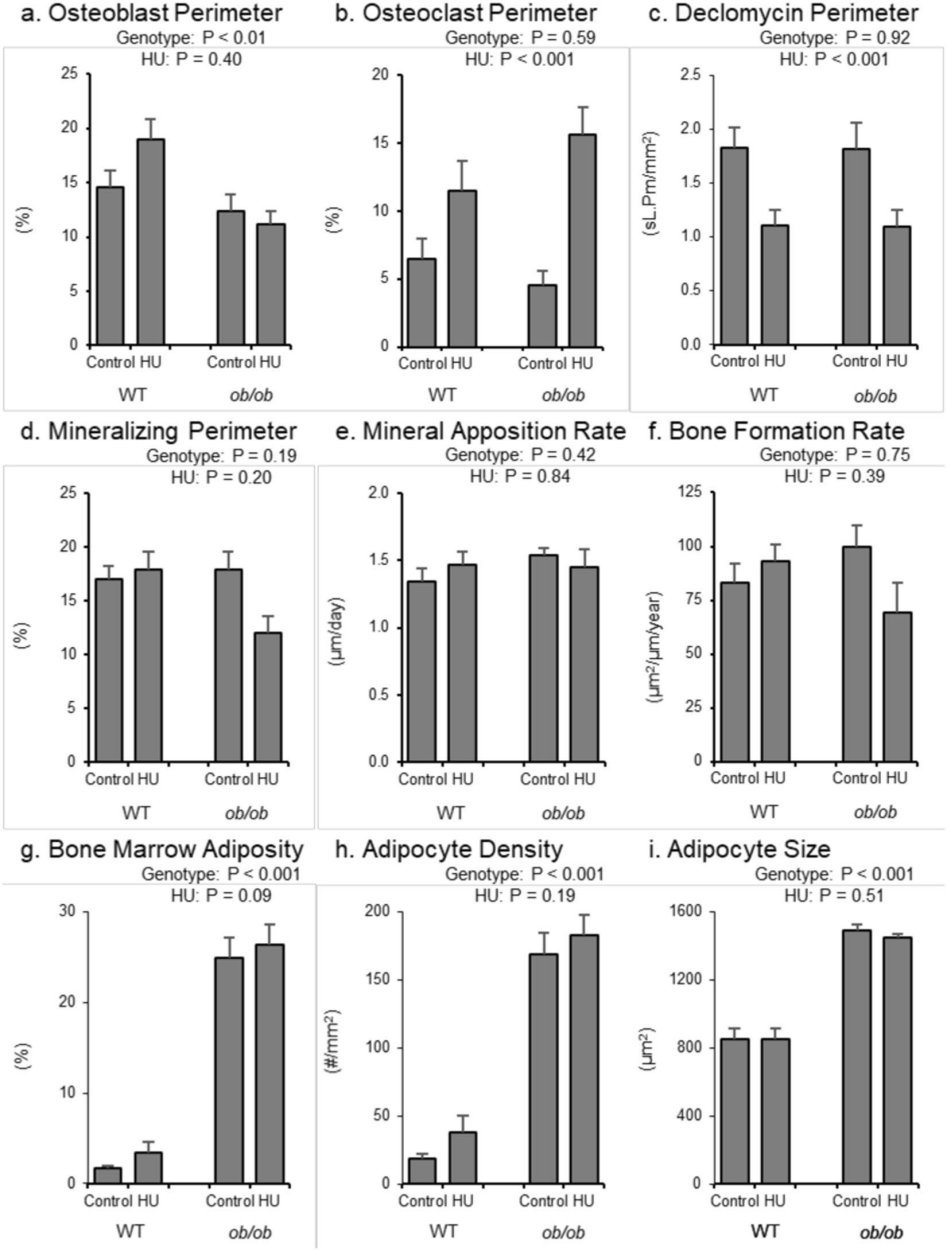
Effects of genotype and hindlimb unloading (HU) on cancellous bone histomorphometry and marrow adiposity in the distal femur metaphysis in WT and *ob/ob* mice. Shown are (**a**) osteoblast perimeter, (**b**) osteoclast perimeter, (**c**) declomycin label retention, (**d**) mineralizing perimeter, (**e**) mineral apposition rate, (**f**) bone formation rate, (**g**) bone marrow adiposity, (**h**) adipocyte density, and (**i**) adipocyte size. Statistical analysis: Two-way ANOVA. P-values for main effects of leptin status (genotype) and skeletal loading status (HU) significant at P ≤ 0.05. Significant (P ≤ 0.05) genotype x HU interactions were not detected with treatment for any of the endpoints evaluated. Mean ± SEM; N = 10/group.

**Figure 8:**
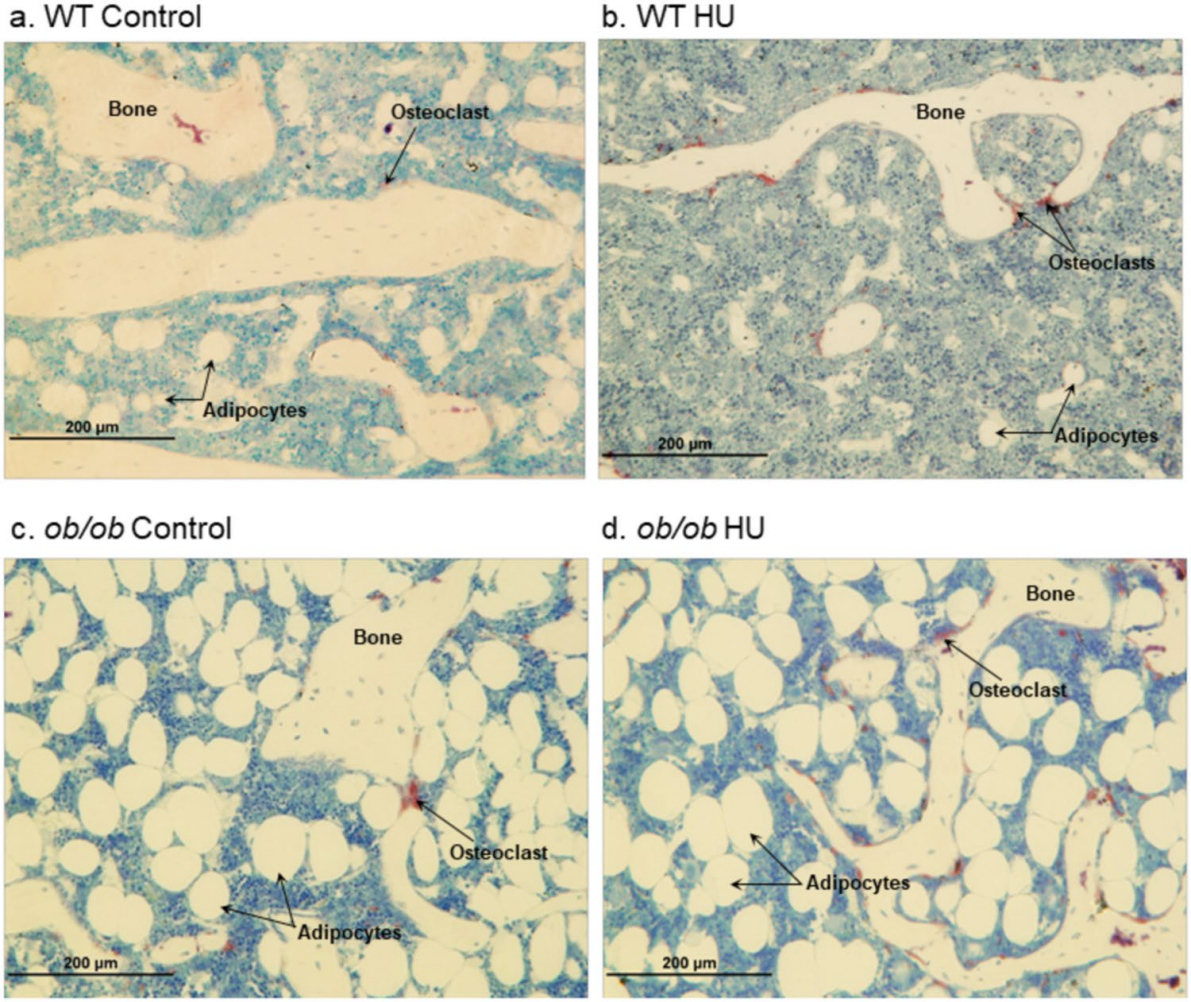
Representative photomicrographs from (**a**) WT control mouse, (**b**) WT HU mouse, (**c**) *ob/ob* control mouse, and (**d**) *ob/ob* HU mouse. Note the higher number of adipocytes in *ob/ob* mice and the higher number of osteoclasts in HU mice. Images taken by RTT.

The respective and combined effects of leptin status and HU on histomorphometric indices of bone formation, bone resorption, and bone marrow adiposity in the distal femur metaphysis are shown in Fig. 7. Osteoblast perimeter (Fig. 7a) was lower in *ob/ob* mice, while osteoclast perimeter (Fig. 7b) and declomycin label length (Fig. 7c) did not differ between genotypes. Mineralizing perimeter (Fig. 7d), mineral apposition rate (Fig. 7e) and bone formation rate (Fig. 7f) did not differ with genotype. *ob/ob* mice had greater bone marrow adiposity (Fig. 7g), adipocyte density (Fig. 7h), and adipocyte size (Fig. 7i) than WT mice. HU resulted in greater osteoclast perimeter and lower declomycin label length. Mineralizing perimeter, mineral apposition rate, and bone formation rate did not differ with HU. No significant difference in adipocyte density or adipocyte size was observed in response to HU, but there was a trend (p = 0.09) for higher bone marrow adiposity. The differences between genotypes in marrow adiposity and osteoclast-lined bone perimeter with HU can be visually appreciated in Fig. 8. No significant genotype by treatment interaction was detected for any of the measured endpoints in distal femur metaphysis.

The respective and combined effects of genotype and HU on cancellous bone microarchitecture in 5^th^ lumbar vertebra are shown in Table 1. Bone volume fraction and trabecular thickness were higher and structure model index was lower in *ob/ob* mice compared to WT mice. HU resulted in lower cancellous bone volume fraction and trabecular thickness, and higher structure model index. However, significant genotype by treatment interactions were noted for trabecular number, trabecular spacing, and connectivity density. HU resulted in lower trabecular number and higher trabecular spacing in WT mice and higher connectivity density in *ob/ob* mice.

**Table 1:** Effects of genotype and hindlimb unloading (HU) on cancellous bone microarchitecture in the 5th lumbar vertebra in WT and *ob/ob* mice.

	WT		*ob/ob*		ANOVA P Value		
	Control	HU	Control	HU	Genotype	HU	Interaction
**5th lumbar vertebra** (cancellous bone)							
Bone Volume/Tissue Volume (%)	22.0 ± 0.3	19.1 ± 0.7	30.4 ± 0.5	27.4 ± 0.9	0.000	0.000	
Connectivity Density (1/mm³)	234.9 ± 5.8	243.5 ± 6.8	220.0 ± 7.4	281.1 ± 13.8^a^			0.012
Structure Model Index	0.8 ± 0.0	1.0 ± 0.1	0.0 ± 0.1	0.2 ± 0.1	0.000	0.001	
Trabecular Number (1/mm)	5.3 ± 0.1	5.0 ± 0.1^a^	5.7 ± 0.1^b^	5.9 ± 0.1^b^			0.006
Trabecular Thickness (µm)	44 ± 0	42 ± 1	52 ± 1	47 ± 1	0.000	0.000	
Trabecular Spacing (µm)	184 ± 2	196 ± 2^a^	167 ± 2^b^	164 ± 3^b^			0.017

Data are mean ± SEM; N = 10/group.

^a^Different from control within genotype, P ≤ 0.05.

^b^Different from WT, P ≤ 0.05.

## Discussion

We evaluated the skeletal response to HU in leptin-deficient *ob/ob* male mice. Based on the observation that leptin deficiency attenuates the positive association between body weight and bone mass (e.g., more weight gain is required in *ob/ob* mice compared to WT mice to achieve a comparable increase in bone mass), we hypothesized that *ob/ob* mice would exhibit diminished bone loss in response to HU. However, contrary to our expectations, the magnitude of HU-induced osteopenia in *ob/ob* mice in femur, a weight bearing bone, was virtually indistinguishable from that of WT mice. In concordance with previous studies in WT mice, cancellous bone loss in HU mice housed at thermoneutral was due to increased bone resorption. The increase in bone resorption was due, at least in part, to increased osteoclast-lined bone perimeter^24^.

Leptin has differential effects on osteoclast number and activity in weight-bearing mice. *ob/ob* mice have normal or increased osteoclast-lined bone perimeter^25,26^, indicating that the hormone is not required for osteoclastogenesis. Indeed, subcutaneous administration of leptin attenuates ovariectomy- and HU-induced bone loss in rats by reducing osteoclast number^3,27^. On the other hand, several lines of evidence suggest reduced osteoclast activity in *ob/ob* mice. Specifically, *ob/ob* mice have low serum levels of CTX, a biochemical marker of global bone resorption^20,26^. In spite of elevated osteoclast-lined bone perimeter, resorption of declomycin is diminished in femurs of *ob/ob* mice^16^. Furthermore, *ob/ob* mice are resistant to polyethylene particle-induced osteolysis^28^. Finally, *ob/ob* mice have impaired ability to remodel the calcified cartilage network in growing femur, leading to pathological retention of cartilage during skeletal maturation^10,16,26^.

In the present study, bone resorption was increased in femur metaphysis in *ob/ob* and WT mice in response to HU. In support, we observed an increase in osteoclast-lined bone perimeter. Furthermore, we observed decreases in cancellous bone volume fraction and fluorochrome label retention. Specifically, declomycin label given at the start of the study was lower after two weeks of HU compared to weight-bearing mice, indicating greater resorption of fluorochrome labeled bone occurred with HU. This association between unloading and label loss is in agreement with previous spaceflight and HU studies in rats^29^. HU for two weeks had no significant effect on body composition in WT or *ob/ob* mice. This is important because changes in weight could independently influence bone turnover balance^30^. Thus, contrary to our expectation, skeletal unloading enhanced bone resorption in leptin-deficient mice.

Reduced bone formation contributes to development of osteopenia in growing rodents during microgravity (spaceflight) and simulated microgravity (HU)^31,32^. However, suppression of bone formation is bone compartment-specific. In HU mice, cortical bone formation is suppressed^33^, but this is not necessarily the case for cancellous skeletal sites^24^. In the present study, osteoblast perimeter and bone formation rate were not reduced in distal femur in WT or *ob/ob* mice during HU. Taken together, these observations suggest that HU results in cancellous bone loss in the distal femur metaphysis because resorption increases to levels that exceed the prevailing level of bone formation.

In addition to femur, we evaluated the respective effects of leptin deficiency and HU on lumbar vertebra. Cancellous bone loss occurs in lumbar vertebra in rodents subjected to microgravity^34^ and the bone loss is due, at least in part, to increased bone resorption^35^. Femurs experience high levels of ground reaction forces in response to weight bearing, and these forces are abolished by HU. Lumbar vertebrae do not generally bear weight in mice. However, lumbar vertebrae are load bearing and HU alters the orientation of the vertebral column to the gravitational vector, changing the direction of the load^36^. Similar to femur, leptin deficiency did not prevent HU-induced cancellous bone loss in lumbar vertebra. While there were no genotype-specific differences in magnitude of cancellous bone loss in lumbar vertebra of HU mice, the bone loss in WT mice was due to a combination of decreased trabecular thickness and trabecular number, whereas the bone loss in *ob/ob* mice was due to decreased trabecular thickness only. Therefore, minor genotype-specific changes in bone microarchitecture accompanied changes in bone volume fraction in lumbar vertebrae following HU.

Spaceflight studies in rodents report lower circulating testosterone levels following spaceflight and ground-based HU studies in rodents report lower seminal vesicle weight^37–42^. In the present study, HU reduced seminal vesicle weight in both genotypes, indicating lower testosterone levels. Lower testosterone levels potentially contribute to elevated bone resorption and cancellous bone loss^43^. However, in spite of gonadal insufficiency, leptin deficiency typically results in a low turnover skeletal phenotype in *ob/ob* mice of both sexes^44^. The precise role of androgens in mediating the action of leptin on bone is unknown but antagonism of estrogen receptor signaling was largely dispensable for leptin’s actions in female *ob/ob* mice^44^. The present results, identifying similar bone loss in HU WT and HU *ob/ob* mice, suggest that preexisting hypogonadism in male *ob/ob* mice did not have a major influence on the magnitude of bone loss following HU.

Compared to WT mice, MAT levels were approximately 10x greater in *ob/ob* mice. In concordance with prior work^15,16,45^, the increase was due to a combination of more numerous and larger adipocytes. HU did not alter adipocyte number or size, findings that contrast with an increase in MAT reported in rats following either spaceflight^46^ or HU^47^ and a previous HU study by Hino *et al*. in male B6 mice^48^. However, the present findings are concordant with a HU study performed in male WBB6F1/J mice housed at thermoneutral^24^. Hino *et al*.^48^ performed their study in 10-week-old mice housed at 24 °C, which is well below thermoneutral. This may be important because sub-thermoneutral housing reduces bone marrow adiposity in B6 mice^49^ and there is accumulating evidence that mild cold stress induced by room temperature housing impacts experimental results^50^.

MAT levels are often (but not always) negatively associated with osteoblast number and bone mass and are increased in humans following long-duration bed rest and in rodents following spaceflight and in some skeletal disuse models^9,24,35,51–55^. Osteoblasts and adipocytes differentiate from bone marrow mesenchymal stem cells (MSCs) and some researchers have concluded that adipocyte differentiation impairs osteoblast differentiation^35^. Furthermore, adipokines produced by MAT have the potential to increase bone resorption^56–58^. Thus, high levels of MAT in mice could theoretically diminish the skeletal response to mechanical loading by at least two non-mutually exclusive mechanisms, impairment of osteoblast differentiation and stimulation of osteoclast activity.

Despite greatly increased levels of MAT in distal femur metaphysis of *ob/ob* mice, the mice did not differ in cancellous bone volume fraction from WT mice. There were also negligible differences between the two genotypes in cortical or cancellous bone responses to HU. Taken together, these findings add to a growing list of studies that contradict a deterministic model where increased MAT levels invariably negatively affect bone metabolism^59^. These findings also fail to support a model where adipokines produced by MAT contribute to HU-induced bone loss.

Cancellous bone volume fraction in femur in *ob/ob* mice has been variably reported to be lower^14^, equivalent (present study), or higher^17^ than in age-matched WT mice. In our experience, cancellous bone volume fraction in the distal femur metaphysis in conventionally-housed (room temperature) mice declines with age in both WT and *ob/ob* mice, but the rate of decline is greater in WT mice. Impaired osteoclast activity in *ob/ob* mice provides a plausible explanation for slower age-related bone loss as well as reported differences in their skeletal phenotype.

Leptin deficiency results in a variety of physical and metabolic changes that could influence osteoclast activity. Metabolically, *ob/ob* mice have impaired thermoregulation, are hypogonadal and hyperphagic, and often have elevated glucose, insulin, and glucocorticoid levels^16^. In the present study, we limited but did not fully eliminate the contribution of metabolic differences by housing *ob/ob* mice at thermoneutral and decreasing their food intake to that of WT mice. *ob/ob* mice preferentially gain fat mass and there is evidence that lean mass contributes more to the positive effect of weight on the skeleton than fat mass^30^. *ob/ob* mice are sarcopenic, and Hamrick and colleagues have reported a strong linear association between bone mineral content and quadriceps mass in *ob/ob* and WT mice^14^. Additional studies are required to evaluate the comparative effect of muscle atrophy on the skeleton of WT and *ob/ob* mice in response to HU.

As expected, leptin deficiency and HU each resulted in higher corticosterone levels. Although glucocorticoids influence bone metabolism, Zerath *et al*.^60^ reported that microgravity-induced cancellous osteopenia occurred independent of endogenous corticosterone secretion. Furthermore, elevation of endogenous corticosterone levels did not replicate the skeletal changes observed in HU rodents^61^. While these two studies do not rule out a role for adrenal hormones, it is important to note that parallel increases in corticosterone with HU were detected in WT and *ob/ob* mice. This is consistent with the similar magnitude of bone loss following HU in the two genotypes.

Skeletal unweighting, whether due to microgravity, bedrest, limb casting, or spinal cord injury results in bone loss^62,63^. Impact loading imparted by ground reaction forces, sensory and sympathetic signaling, surgery, and muscle loading each influence bone metabolism^64–67^. Commonly used models for unweighting the skeleton include HU, unilateral sciatic neurotomy, and limb casting. HU was originally designed as an earth-based model for microgravity^68^ and chosen for the current study because (1) it is minimally invasive (i.e., does not require surgery), (2) prevents weight bearing, but unlike limb casting does not prohibit voluntary muscle loading, and (3) in contrast to unilateral sciatic neurotomy, does not directly influence neuronal regulation of bone metabolism.

Leptin is a potent stimulator of bone accrual, but the positive effects of leptin on bone formation in mice occur at low circulating levels of the hormone^69^. Similar to mice, weight gain in humans is generally associated with increases in BMD^30^. However, obesity results in hyperleptinemia and the positive association between increased body weight and increased BMD is attenuated in obese subjects^70^. Prolonged bed rest, a model for skeletal unloading in humans, results in rapid bone loss^71^. Bedrest studies are typically performed in healthy individuals but bedrest is sometimes used in management of hospitalized patients diagnosed with anorexia nervosa. These individuals, who have very low leptin levels, appear to also exhibit unbalanced bone turnover leading to bone loss^72,73^.

There are limitations to the present study. The study focused on the role of leptin in skeletal response to changes in weight. While impact loading on the hindlimbs caused by weight is important to skeletal health, we cannot rule out that leptin plays a role in other forms of skeletal loading, such as loading mediated through muscle contraction. Furthermore, we performed the present study in male mice. Since estrogen is important in regulation of appetite, energy expenditure and metabolism, future studies comparing the skeletal response of female *ob/ob* and WT HU mice are warranted.

In summary, chronic leptin deficiency in male *ob/ob* mice resulted in shorter femora, lower femoral bone mass and density, alterations in cortical bone microarchitecture, site- and bone-specific alterations in cancellous bone architecture, and greatly increased MAT levels. These results indicate that leptin plays a critical role in normal bone growth, maturation, and turnover. Contrary to expectation, leptin deficiency did not alter the magnitude of HU-induced osteopenia in the mouse femur. These findings suggest that leptin is not essential for the skeletal response to unweighting.

## Methods

The animals were maintained in accordance with the NIH Guide for the Care and Use of Laboratory Animals and the Oregon State University Institutional Animal Care and Use Committee approved the experimental protocol.

Four-week-old male C57BL/6J (WT, n = 20) mice and B6.Lep^ob/ob^ (*ob/ob*, n = 20) mice were purchased from Jackson Laboratory (Bar Harbor, ME, USA) and single-housed in a 32 °C room for the duration of the experiment. Housing mice at 32 °C (thermoneutral temperature) minimizes resting energy expenditure^74,75^ and prevents premature cancellous bone loss associated with cold stress induced by sub-thermoneutral housing^49^. Water was provided *ad libitum* to all animals for the duration of study. *ob/ob* mice were pair-fed to WT mice from 4 to 16 weeks of age to minimize differences in weight gain^16^. At 16 weeks of age, the mice were randomized by body weight into one of four treatment groups (n = 10/group): (1) WT control, (2) WT HU, (3) *ob/ob* control, and (4) *ob/ob* HU. Immediately prior to HU, animals were given a declomycin injection (15 mg/kg; sc). The mice were unloaded for 2 weeks as described^21^. In brief, HU mice were placed in a restraint device, where the tail was cleaned with ethanol-soaked gauze and sprayed with a tincture of benzoin. A thin piece of traction tape was looped through a large paper clip, and then pressed along the sides of the mouse’s tail. Filament tape was wrapped around the tail in two locations to secure the traction tape: the base of the tail and 2.5 cm caudal. The paperclip end was looped through the clasp secured on the unloading apparatus. Mice were positioned in a 30° head-down tilt. During unloading, the *ob/ob* HU group and control groups of both genotypes were pair-fed to the WT HU group. The fluorochrome calcein (15 mg/kg; sc) was administered at 4 days and 1 day prior to sacrifice. Mice were anesthetized with isoflurane and terminated using decapitation. Blood was collected from the trunk following decapitation. Serum was stored at −80 °C. Femora and 5^th^ lumbar vertebrae from each mouse were placed in formalin for 24-hour fixation, then stored at 4 °C in 70% ethanol. Body weight (g), abdominal WAT weight (g) and seminal vesicle weight (g) were recorded at necropsy.

### Blood chemistry

Blood glucose was measured using a glucometer (Life Scan, Inc., Milpitas, CA, USA) immediately following decapitation. Serum corticosterone (ng/ml) and osteocalcin (ng/ml) were measured using a mouse Corticosterone ELISA kit obtained from Abcam (Cambridge, MA, USA) and a mouse Gla-osteocalcin High Sensitive EIA kit obtained from Clontech (Takara Bio Inc Shiga, Japan), respectively.

### Densitometry

Total femur bone mineral content (BMC, g), bone area (cm^2^), and bone mineral density (BMD, g/cm^2^) were measured using dual energy x-ray absorptiometry (DXA, Piximus 2, Lunar Corporation, Madison, WI, USA).

### Micro-computed tomography

µCT was used for nondestructive three-dimensional evaluation of total femur bone volume (mm^3^), femur length (mm), and cortical and cancellous bone architecture. Femora were scanned using a Scanco µCT40 scanner (Scanco Medical AG, Basserdorf, Switzerland) at a voxel size of 12 µm × 12 µm × 12 µm (55 kV_p_ x-ray voltage, 145 µA intensity, and 200 ms integration time). Filtering parameters sigma and support were set to 0.8 and 1, respectively. The threshold value for evaluation was determined empirically and set at 245 (gray scale, 0–1000). Cortical bone was evaluated in the femoral diaphysis and cancellous bone was evaluated in the distal femur metaphysis and epiphysis (Fig. 3).

Assessment of cortical bone in the femur diaphysis began 60% down the midshaft from the femoral head and consisted of 20 slices (240 µm in length). Automated contouring was used to delineate cortical bone from the marrow cavity. All cortical slices were visually examined for evidence of cancellous struts originating from the endocortex and manually removed when present. Direct cortical bone measurements included total cross-sectional volume (mm^3^), cortical volume (mm^3^), marrow volume (mm^3^), and cortical thickness (µm). Polar moment of inertia (mm^4^) was determined as a surrogate measure of bone strength in torsion.

Assessment of cancellous bone in the distal femur metaphysis began 45 slices (540 µm in length) proximal to the growth plate, and included 40 slices (480 µm in length) of cancellous bone. The entire cancellous bone compartment was evaluated in the distal femur epiphysis (31 ± 1 slices) and 5^th^ lumbar vertebral body (149 ± 1 slices). Direct cancellous bone measurements included bone volume fraction (bone volume/tissue volume; volume of total tissue occupied by cancellous bone, %), connectivity density (number of redundant connections per unit volume, 1/mm^3^), structure model index (an architectural index defining bone as plate-like or rod-like with values ranging from 0 to 3, respectively), trabecular thickness (mean thickness of individual trabeculae, µm), trabecular number (number of trabecular intercepts per unit length, 1/mm) and trabecular spacing (distance between trabeculae, µm).

### Histomorphometry

The histological methods used have been previously described in detail^76^. In brief, distal femora were dehydrated in graded increases of ethanol and xylene, then embedded undecalcified in methyl methacrylate. Sections, 4 µm thick, were cut with a vertical bed microtome (Leica/Jung 2165) and affixed to slides with a dried pre-coated 1% gelatin solution. Slides were stained for tartrate-resistant acid phosphatase and counterstained with toluidine blue (Sigma, St. Louis, MO, USA) and used for cell-based measurements. Mounted unstained slides were used for measurements of fluorochrome labels. All data were collected using the OsteoMeasure System (OsteoMetrics, Inc., Atlanta, GA, USA). The sampling site for the distal femoral metaphysis was located 0.25–1.25 mm proximal to the growth plate.

Static (cell-based) histological measurements include osteoblast perimeter (osteoblast perimeter/bone perimeter; %), osteoclast perimeter (osteoclast perimeter/bone perimeter; %), bone marrow adiposity (adipocyte area/tissue area; %), adipocyte density (#/mm^2^), and adipocyte size (µm^2^). Osteoblast perimeter was determined as a percentage of total bone perimeter lined by cuboidal cells adjacent to a thin layer of osteoid in direct physical contact with bone. Osteoclast perimeter was determined as a percentage of cancellous bone perimeter covered by multinucleated cells with an acid phosphatase-positive (stained red) cytoplasm. Adipocytes were identified as large circular or oval-shaped cells bordered by a prominent cell membrane lacking cytoplasmic staining due to alcohol extraction of intracellular lipids during processing^77^. Fluorochrome-based measurements of cancellous bone formation included mineralizing perimeter (mineralizing perimeter/bone perimeter: perimeter covered with double label plus half single label, normalized to bone perimeter, %), mineral apposition rate (the distance between double calcein labels, divided by the 3 day interlabel period, µm/day), and bone formation rate (mineralizing perimeter multiplied by mineral apposition rate expressed per bone perimeter (µm^2^/µm/y).

Retained declomycin label (single label perimeter/tissue area; mm/mm^2^) in the distal femur metaphysis was measured as a dynamic index of bone resorption. This method is based on the premise that treatment groups did not differ in bone formation (and as such fluorochrome label incorporation) prior to HU^35^ and that differences measured at the study termination reflect the effect of treatment on resorption of the fluorochrome-labeled bone^78^.

### Statistical analysis

Means were compared between genotype and treatment groups using two-way analysis of variance (ANOVA). When significant interactions were present, t-tests were used to make two-group comparisons. When non-significant interactions were present, group comparisons were made from two-way ANOVA with main effects for genotype and treatment. The required conditions for valid use of t-tests and ANOVA were assessed using Levene’s test for homogeneity of variance and the Anderson-Darling test of normality. When the assumption of equal variance was violated, Welch’s two-sample t-test was used for two-group comparisons^79^. When the normality assumption was violated, the Wilcoxon-Mann-Whitney test was used for two-group comparisons. Methods for maintaining false discovery rate at 5% were used to adjust for multiple comparisons^80^. Differences were considered significant at p ≤ 0.05. Data are presented as mean ± SEM. Data analysis was performed using RStudio version 0.98.1083.

## Supplementary information

Supplemental Figure S1

## Data Availability

Data in the manuscript has been archived with NASA and will be publically available.

## References

[1] Mistry AM, Swick AG, Romsos DR (1997). Leptin Rapidly Lowers Food Intake and Elevates Metabolic Rates in Lean and Ob/Ob Mice. J Nutr.

[2] Myers MG (2004). Leptin Receptor Signaling and the Regulation of Mammalian Physiology. Recent Prog Horm Res.

[3] Burguera B (2001). Leptin Reduces Ovariectomy-Induced Bone Loss in Rats. Endocrinology.

[4] Thomas T (2004). The Complex Effects of Leptin on Bone Metabolism through Multiple Pathways. Curr Opin Pharmacol.

[5] Gordeladze JO, Drevon CA, Syversen U, Reseland JE (2002). Leptin Stimulates Human Osteoblastic Cell Proliferation, De Novo Collagen Synthesis, and Mineralization: Impact on Differentiation Markers, Apoptosis, and Osteoclastic Signaling. J Cell Biochem.

[6] Reseland JE (2001). Leptin Is Expressed in and Secreted from Primary Cultures of Human Osteoblasts and Promotes Bone Mineralization. J Bone Miner Res.

[7] Reid IR, Baldock PA, Cornish J (2018). Effects of Leptin on the Skeleton. Endocr Rev.

[8] Steppan CM, Crawford DT, Chidsey-Frink KL, Ke H, Swick AG (2000). Leptin Is a Potent Stimulator of Bone Growth in Ob/Ob Mice. Regul Pept.

[9] Hamrick MW (2005). Leptin Treatment Induces Loss of Bone Marrow Adipocytes and Increases Bone Formation in Leptin-Deficient Ob/Ob Mice. J Bone Miner Res.

[10] Philbrick KA (2018). Effects of Hypothalamic Leptin Gene Therapy on Osteopetrosis in Leptin-Deficient Mice. J Endocrinol.

[11] Yagasaki Y (2003). The Role of Craniofacial Growth in Leptin Deficient (Ob/Ob) Mice. Orthod Craniofac Res.

[12] Dubuc PU (1976). The Development of Obesity, Hyperinsulinemia, and Hyperglycemia in Ob/Ob Mice. Metabolism.

[13] Smith CK, Romsos DR (1984). Cold Acclimation of Obese (Ob/Ob) Mice: Effects on Skeletal Muscle and Bone. Metabolism.

[14] Hamrick MW, Pennington C, Newton D, Xie D, Isales C (2004). Leptin Deficiency Produces Contrasting Phenotypes in Bones of the Limb and Spine. Bone.

[15] Bartell SM (2011). Central (Icv) Leptin Injection Increases Bone Formation, Bone Mineral Density, Muscle Mass, Serum Igf-1, and the Expression of Osteogenic Genes in Leptin-Deficient Ob/Ob Mice. J Bone Miner Res.

[16] Turner RT (2014). Morbid Obesity Attenuates the Skeletal Abnormalities Associated with Leptin Deficiency in Mice. J Endocrinol.

[17] Iwaniec UT, Boghossian S, Lapke PD, Turner RT, Kalra SP (2007). Central Leptin Gene Therapy Corrects Skeletal Abnormalities in Leptin-Deficient Ob/Ob Mice. Peptides.

[18] Kapur S (2010). Leptin Receptor (Lepr) Is a Negative Modulator of Bone Mechanosensitivity and Genetic Variations in Lepr May Contribute to the Differential Osteogenic Response to Mechanical Stimulation in the C57bl/6j and C3h/Hej Pair of Mouse Strains. J Biol Chem.

[19] Iwaniec UT (2009). Body Mass Influences Cortical Bone Mass Independent of Leptin Signaling. Bone.

[20] Philbrick KA, Turner RT, Branscum AJ, Wong CP, Iwaniec UT (2015). Paradoxical Effects of Partial Leptin Deficiency on Bone in Growing Female Mice. Anat Rec (Hoboken).

[21] Morey-Holton ER, Globus RK (2002). Hindlimb Unloading Rodent Model: Technical Aspects. J Appl Physiol (1985).

[22] Iwaniec UT (2016). Room Temperature Housing Results in Premature Cancellous Bone Loss in Growing Female Mice: Implications for the Mouse as a Preclinical Model for Age-Related Bone Loss. Osteoporos Int.

[23] Gavrilova O (1999). Torpor in Mice Is Induced by Both Leptin-Dependent and -Independent Mechanisms. Proc Natl Acad Sci USA.

[24] Keune JA, Wong CP, Branscum AJ, Iwaniec UT, Turner RT (2017). Bone Marrow Adipose Tissue Deficiency Increases Disuse-Induced Bone Loss in Male Mice. Sci Rep.

[25] Agarwal S (2015). Diminished Chondrogenesis and Enhanced Osteoclastogenesis in Leptin-Deficient Diabetic Mice (Ob/Ob) Impair Pathologic, Trauma-Induced Heterotopic Ossification. Stem Cells Dev.

[26] Turner RT (2013). Peripheral Leptin Regulates Bone Formation. J Bone Miner Res.

[27] Baek K, Bloomfield SA (2009). Beta-Adrenergic Blockade and Leptin Replacement Effectively Mitigate Disuse Bone Loss. J Bone Miner Res.

[28] von Knoch M (2004). Decrease in Particle-Induced Osteolysis in Obese (Ob/Ob) Mice. Biomaterials.

[29] Hefferan TE (2003). Effect of Gender on Bone Turnover in Adult Rats During Simulated Weightlessness. J Appl Physiol (1985).

[30] Iwaniec UT, Turner RT (2016). Influence of Body Weight on Bone Mass, Architecture and Turnover. J Endocrinol.

[31] Turner RT (2000). Invited Review: What Do We Know About the Effects of Spaceflight on Bone?. J Appl Physiol (1985).

[32] Globus RK, Bikle DD, Morey-Holton E (1986). The Temporal Response of Bone to Unloading. Endocrinology.

[33] Bateman TA, Broz JJ, Fleet ML, Simske SJ (1997). Differing Effects of Two-Week Suspension on Male and Female Mouse Bone Metabolism. Biomed Sci Instrum.

[34] Keune JA, Branscum AJ, Iwaniec UT, Turner RT (2015). Effects of Spaceflight on Bone Microarchitecture in the Axial and Appendicular Skeleton in Growing Ovariectomized Rats. Sci Rep.

[35] Keune JA, Philbrick KA, Branscum AJ, Iwaniec UT, Turner RT (2016). Spaceflight-Induced Vertebral Bone Loss in Ovariectomized Rats Is Associated with Increased Bone Marrow Adiposity and No Change in Bone Formation. Npj Microgravity.

[36] Ogneva IV, Biryukov NS (2013). Mathematical Modeling of Cardiomyocytes’ and Skeletal Muscle Fibers’ Membrane: Interaction with External Mechanical Field. Applied Mathematics.

[37] Amann RP (1992). Effects of Microgravity or Simulated Launch on Testicular Function in Rats. J Appl Physiol (1985).

[38] Grindeland RE, Popova IA, Vasques M, Arnaud SB (1990). Cosmos 1887 Mission Overview: Effects of Microgravity on Rat Body and Adrenal Weights and Plasma Constituents. FASEB J.

[39] Merrill AH, Wang E, Mullins RE, Grindeland RE, Popova IA (1992). Analyses of Plasma for Metabolic and Hormonal Changes in Rats Flown Aboard Cosmos 2044. J Appl Physiol (1985).

[40] Strollo F (1998). The Effect of Microgravity on Testicular Androgen Secretion. Aviat Space Environ Med.

[41] Tash JS, Johnson DC, Enders GC (2002). Long-Term (6-Wk) Hindlimb Suspension Inhibits Spermatogenesis in Adult Male Rats. J Appl Physiol (1985).

[42] De Naeyer H (2015). Effects of Tail Suspension on Serum Testosterone and Molecular Targets Regulating Muscle Mass. Muscle Nerve.

[43] Wiren KM, Zhang XW, Olson DA, Turner RT, Iwaniec UT (2012). Androgen Prevents Hypogonadal Bone Loss Via Inhibition of Resorption Mediated by Mature Osteoblasts/Osteocytes. Bone.

[44] Turner RT, Philbrick KA, Kuah AF, Branscum AJ, Iwaniec UT (2017). Role of Estrogen Receptor Signaling in Skeletal Response to Leptin in Female Ob/Ob Mice. J Endocrinol.

[45] Lindenmaier LB (2016). Hypothalamic Leptin Gene Therapy Reduces Bone Marrow Adiposity in Ob/Ob Mice Fed Regular and High Fat Diets. Front Endocrinol (Lausanne).

[46] Jee WS, Wronski TJ, Morey ER, Kimmel DB (1983). Effects of Spaceflight on Trabecular Bone in Rats. Am J Physiol.

[47] Ahdjoudj S, Lasmoles F, Holy X, Zerath E, Marie PJ (2002). Transforming Growth Factor Beta2 Inhibits Adipocyte Differentiation Induced by Skeletal Unloading in Rat Bone Marrow Stroma. J Bone Miner Res.

[48] Hino K (2006). Unloading-Induced Bone Loss Was Suppressed in Gold-Thioglucose Treated Mice. J Cell Biochem.

[49] Iwaniec, U. T. *et al*. Room Temperature Housing Results in Premature Cancellous Bone Loss in Growing Female Mice: Implications for the Mouse as a Preclinical Model for Age-Related Bone Loss. *Osteoporosis International* **[**Advance Publication**]** (2016). 10.1007/s00198-016-3634-3 PMC5421618 27189604

[50] Hylander BL, Repasky EA (2016). Thermoneutrality, Mice, and Cancer: A Heated Opinion. Trends Cancer.

[51] Li M, Liang H, Shen Y, Wronski TJ (1999). Parathyroid Hormone Stimulates Cancellous Bone Formation at Skeletal Sites Regardless of Marrow Composition in Ovariectomized Rats. Bone.

[52] Martin RB, Zissimos SL (1991). Relationships between Marrow Fat and Bone Turnover in Ovariectomized and Intact Rats. Bone.

[53] Muruganandan S, Sinal CJ (2014). The Impact of Bone Marrow Adipocytes on Osteoblast and Osteoclast Differentiation. IUBMB Life.

[54] Verma S, Rajaratnam JH, Denton J, Hoyland JA, Byers RJ (2002). Adipocytic Proportion of Bone Marrow Is Inversely Related to Bone Formation in Osteoporosis. J Clin Pathol.

[55] Turner RT, Martin SA, Iwaniec UT (2018). Metabolic Coupling between Bone Marrow Adipose Tissue and Hematopoiesis. Curr Osteoporos Rep.

[56] Thommesen L (2006). Expression and Regulation of Resistin in Osteoblasts and Osteoclasts Indicate a Role in Bone Metabolism. J Cell Biochem.

[57] Shimizu H (2008). Angiotensin Ii Accelerates Osteoporosis by Activating Osteoclasts. FASEB J.

[58] Tu Z, Bu H, Dennis JE, Lin F (2010). Efficient Osteoclast Differentiation Requires Local Complement Activation. Blood.

[59] Akune T (2004). Ppargamma Insufficiency Enhances Osteogenesis through Osteoblast Formation from Bone Marrow Progenitors. J Clin Invest.

[60] Zerath E (2000). Spaceflight Inhibits Bone Formation Independent of Corticosteroid Status in Growing Rats. J Bone Miner Res.

[61] Li M (1996). Skeletal Response to Corticosteroid Deficiency and Excess in Growing Male Rats. Bone.

[62] Giangregorio L, Blimkie CJ (2002). Skeletal Adaptations to Alterations in Weight-Bearing Activity: A Comparison of Models of Disuse Osteoporosis. Sports Med.

[63] Qin W, Bauman WA, Cardozo C (2010). Bone and Muscle Loss after Spinal Cord Injury: Organ Interactions. Ann N Y Acad Sci.

[64] Hill EL, Turner R, Elde R (1991). Effects of Neonatal Sympathectomy and Capsaicin Treatment on Bone Remodeling in Rats. Neuroscience.

[65] Iwaniec UT (2011). Hypothalamic Leptin Gene Therapy Prevents Weight Gain without Long-Term Detrimental Effects on Bone in Growing and Skeletally Mature Female Rats. J Bone Miner Res.

[66] Reginster JY, Beaudart C, Buckinx F, Bruyere O (2016). Osteoporosis and Sarcopenia: Two Diseases or One?. Curr Opin Clin Nutr Metab Care.

[67] Turner RT, Wakley GK, Szukalski BW (1985). Effects of Gravitational and Muscular Loading on Bone Formation in Growing Rats. Physiologist.

[68] Morey ER, Sabelman EE, Turner RT, Baylink DJ (1979). A New Rat Model Simulating Some Aspects of Space Flight. Physiologist.

[69] Philbrick KA, Wong CP, Branscum AJ, Turner RT, Iwaniec UT (2017). Leptin Stimulates Bone Formation in Ob/Ob Mice at Doses Having Minimal Impact on Energy Metabolism. J Endocrinol.

[70] Nielson CM, Srikanth P, Orwoll ES (2012). Obesity and Fracture in Men and Women: An Epidemiologic Perspective. J Bone Miner Res.

[71] Hargens AR, Vico L (2016). Long-Duration Bed Rest as an Analog to Microgravity. J Appl Physiol (1985).

[72] DiVasta AD, Feldman HA, Quach AE, Balestrino M, Gordon CM (2009). The Effect of Bed Rest on Bone Turnover in Young Women Hospitalized for Anorexia Nervosa: A Pilot Study. J Clin Endocrinol Metab.

[73] Premaor MO, Pilbrow L, Tonkin C, Parker RA, Compston J (2010). Obesity and Fractures in Postmenopausal Women. J Bone Miner Res.

[74] Trayhurn P (1979). Thermoregulation in the Diabetic-Obese (Db/Db) Mouse. The Role of Non-Shivering Thermogenesis in Energy Balance. Pflugers Arch.

[75] Swoap SJ, Gutilla MJ (2009). Cardiovascular Changes During Daily Torpor in the Laboratory Mouse. Am J Physiol Regul Integr Comp Physiol.

[76] Iwaniec UT, Wronski TJ, Turner RT (2008). Histological Analysis of Bone. Methods Mol Biol.

[77] Menagh PJ (2010). Growth Hormone Regulates the Balance between Bone Formation and Bone Marrow Adiposity. J Bone Miner Res.

[78] Westerlind KC (1997). Estrogen Regulates the Rate of Bone Turnover but Bone Balance in Ovariectomized Rats Is Modulated by Prevailing Mechanical Strain. Proc Natl Acad Sci USA.

[79] Welch B (1951). On the Comparison of Several Mean Values: An Alternative Approach. Biometrika.

[80] Benjamini Y, Hochberg Y (1995). Controlling the False Discovery Rate: A Practical and Powerful Approach to Multiple Testing. J Royal Statistical Society Series B.

