# Supplementary material for: Effect of Leptin Deficiency on the Skeletal Response to Hindlimb Unloading in Adult Male Mice
Source: Sci Rep. 2019 Jun 27;9:9336. doi: 10.1038/s41598-019-45587-0 (PMC6597714; doi:10.1038/s41598-019-45587-0)
Supplement: Supplementary file 1 — Supplemental Figure S1 [file 41598_2019_45587_MOESM1_ESM.pdf]

# Effect of Leptin Deficiency on the Skeletal Response to Hindlimb Unloading in Adult Male Mice

Jessica A. Keune[a], Adam J. Branscum[b], Carmen P. Wong[a], Urszula T. Iwaniec[a,c], and Russell T. Turner[a,c]*

[a]Skeletal Biology Laboratory, School of Biological and Population Health Sciences, Oregon State University, Corvallis, OR 97331, USA

[b]Biostatistics Program, School of Biological and Population Health Sciences, Oregon State University, Corvallis, OR 97331, USA

[c]Center for Healthy Aging Research, Oregon State University, Corvallis, OR 97331, USA

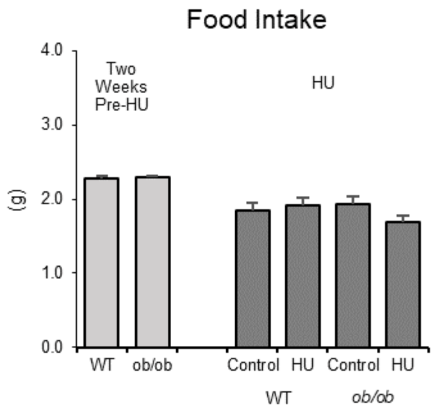


**Supplemental Figure S1**. Food intake by genotype two weeks prior to hindlimb unloading (HU) initiation (*ob/ob* mice pair fed to WT mice) and during the two weeks of HU (all mice pair fed to HU WT mice). Food intakes were compared during HU by group using ANOVA for interaction effects. Since there was no significant interaction term, ANOVA for main effects was performed. There was no significant effect of treatment ($p=0.80$) or genotype ($p=0.85$). Mean ± SEM; N = 10/group.
